# A Treatise on Diseases of the Breast, Both Simple and Cancerous

**Published:** 1846-07

**Authors:** 


### Art. XII.

*Traité des Maladies du Sein, comprenant ses Affections simples et cancéreuses.* Par J. Carpentier-Méricourt, m.d.—*Paris,* 1845.

*A Treatise on Diseases of the Breast, both simple and cancerous.* By J. Carpentier-Méricourt, m.d.—*Paris,* 1845. 8vo, pp. 312.

This book professes to contain a practical exposition of the pathology and treatment of all the affections to which the breast, in females and males, is liable. It is sufficiently plain and unpretending in character, and contains scarcely a single fact unknown to the well-read in the literature of these diseases; yet the volume may claim the merit of presenting a compendious and concise account of the subjects with which it deals. From place to place we find a passage for some motive or other worth notice; and of such passages we proceed to give brief intimation.

*Number of mammæ.* Numerous cases of anomaly of number of the female breast have been reported by writers in this country; but we were not aware that the unfortunate Anne Boleyn (wife of Henry VIII), had three mammæ, and six fingers to each hand, until we found the statement in M. Méricourt's compilation.

*Sore nipples.* The severe ulcerations produced in the nipple of nurses from suckling an infant affected with syphilis, may, the author says, "undergo cancerous degeneration, if they be kept up by stimulant regimen or ill-directed treatment, and if *the female be predisposed to cancer.*" This we believe to be a correct statement; but it would have been well had the author added, that in point of actual fact the necessary combination is extremely rare, inasmuch the origin of cancerous ulceration of the breast in a syphilitic sore is itself extremely rare. His notions concerning the origin of cancer are not always so just however; for we find him elsewhere (p. 30) talking of "little *lipomatous tumours* degenerating into *cancers.*"

Not a work reaches us from Paris without exhibiting fresh proofs of the literary ignorance of the French: here we find this author talking of "MM. Tanchou and Eguisier" as the persons who have drawn attention to kiestein in the urine as a sign of pregnancy!

*Swelling of the mammæ.* When swelling of the breasts follows suppression of the menstrual discharge, itself dependent on the formation of tumours in the uterus, secretion of milk has, according to M. Méricourt, been known to occur. We have not met with any example of the fact, and wish the writer had furnished us with his authority for the statement. He insists much on the practical importance of a knowledge of the "sympathies of the breasts," as treatment may sometimes be effectually directed on the indications thus furnished. He observes, "one of the best methods that can be employed for the suppression of uterine hemorrhage consists in the application of large cupping-glasses to the breasts, so as to determine a salutary fluxion towards those organs." We have no experience of the practice; but can conceive its being useful as an *auxiliary.*

*Hypertrophy of the mammæ.* Turning to the section on hypertrophy of the breast we find another example of the combined ignorance and petty larceny, which it grieves us to be so often obliged to notice in the productions of the Parisian press. M. C. Méricourt divides hypertrophies of the mammæ into two classes,—those of hypertrophy of rather acute course, coinciding with the establishment of menstruation, and those of slow course, connected with certain disorders in the functions of the uterus. The merit of this division (such as it is) he attributes to "M. Aug. Bérard," who used it in "the remarkable essay which signalized his admission into the Faculty of Medicine as Professor of Clinical Surgery." But the truth is that the Professor of Clinical Surgery is no more the author of the arrangement of these diseases than the man in the moon ; the arrangement is appropriated or adopted from the essay of Fingerhuth, which said Fingerhuth's essay, be it observed, M. Méricourt refers to more than once.

*Milk abscess.* In describing the treatment of milk abscess the author avows himself in strong terms a partisan of early opening with the lancet, no matter how deeply seated the suppuration may be : he conceives that delay leads to the risk of "exterior ravages" on the part of the pus. He is well aware that some surgeons do not interfere until the purulent fluid has actually made its way to the skin ; and that others hold that these abscesses should be allowed to open spontaneously. He admits that by following his plan several abscesses may require to be opened one after another, but maintains that there is an inconvenience much less serious than the risk incurred of ulterior injury to the mammæ by the undermining and destructive influence of the accumulating pus. "I do not conceal from myself," adds the writer, "that it requires a deeply-felt conviction to induce one to act in this manner; for patients, more prone to ingratitude than to the contrary, are ready to believe that their medical attendant is to blame, if several abscesses appear, and require opening, one after another; they accuse him either of having allowed all these successive accumulations to take place, or of having opened the first badly." We cannot too highly commend the ethics of M. Méricourt which lead him to prefer what he conceives to be the real advantage of the patient, to the furtherance of his own interests in families. But we differ from him on the point of practice; we are advocates of the single late incision (this is not a case in which a "stitch in time saves nine,") and have not seen the mischiefs arise which he appears so seriously to apprehend, though we are aware such have sometimes occurred. He speaks of numerous incisions into the breasts, as though no physical suffering or danger of any kind attended their performance,—a conclusion to which the incised woman, on the one hand, and the records of unfortunate cases, on the other, would offer very strong demurrer.

*Erectile tumours.* Under the head of erectile tumours of the breast the author relates from a French journal a very singular case (no matter how the narration be understood), of which the following may be taken as a condensed account. A woman lost her infant four days after its birth ; she then had her breasts suckled by a new-born lamb. After the lapse of a few days the latter appeared ill, and violet stains, like flea-bites, appeared on its lips. Subsequently on the seats of discoloration small mammillated fungous excrescences, bleeding on the slightest touch, grew up. Under the continued application of vinegar, oil, and salt, mixed together, these little fungi blackened and fell off. The animal used the woman's breast for a month. But the curious part of the story is that after the suckling had been going forward for a fortnight, and the animal had been ill for a week, the woman perceived on the areola of the left breast some spots of violet colour, from the centre of which sprang seven little bodies about the size of a pin's head. These rapidly became fungous, enlarged, and bled on the slightest injury. On the 17th of January (three weeks after the loss of the infant) three other little pimples appeared on the *right* breast; on the 24th, the little productions on the *left* breast equalled a pear in size. Leeches, compression, the application of calcined alum, opiate cerates, &c. were employed, but to no purpose; indeed the fungi on the *right* breast, uninterfered with, had increased much less rapidly. On the 9th February, M. Nozaran, of Montpellier, found a cauliflower-shaped mass, about 43 lines broad at the broadest part, and 20 at the pedicle, formed chiefly of veins, but containing some minute arteries and nervous filaments. The patient's general health was good; but the local disease visibly increased day by day. A ligature, and next the Vienna caustic were applied; the mass on the right breast disappeared under these means; that on the left resisted them, and was eventually cut out, cicatrization following rapidly. The case as we said is curious; but our interpretation of the facts would not accord with that of M. Méricourt. The vegetations were to our minds probably syphilitic in origin, and the woman tainted the lamb, and not the lamb the woman. The application of the term "erectile tumours" to the excrescences is in keeping with the vague manner in which the term is constantly used; no proof exists that a particle of true erectile tissue existed in the mass.

*Neuralgia.* We turned to the writer's chapter on neuralgia of the breast with some interest, hoping (perhaps we had no right to hope anything of the kind, for how many have laboured in vain on the subject) that some novel and useful suggestion might present itself for the treatment of those obstinate affections. We have been baffled in our own practice in affording anything but temporary relief in cases when every method ever dreamed of was, we conscientiously believe, employed before cure was despaired of,—with the exception of subcutaneous incisions and *amputation of the breast*. Nor are we disposed to have recourse to either of these practices. Although improvement has occurred from the former, it has neither (as far as we are acquainted with the evidence) been more permanent or more complete than from acupuncturation or even the endermic application of morphia. And with respect to amputation of the breast, which has been performed by M. Rufz of Martinique at the eager solicitation of a sufferer from the disease, we hold it to be utterly unwarrantable, unless the operation have been decided as indicated in *consultation*—an infinitely unlikely occurrence. We do not believe that this affection is by any means so frequently connected with that state, unknown in its real nature, to which the name of spinal irritation is commonly given, as it is the fashion of clinical teachers to assume and affirm.

Fragments of fœtus, hair, bone, &c. have in excessively rare instances been found in tumours in the breast; and the mode of their first discovery by the patient is of interesting practical significance. Though these tumours *must* in part, from the nature of things be congenital, yet they have not in the few known cases attracted attention until accidental circumstances (a blow, a fall, &c.) have given an impulse to the growth of the solid matter connected with them. They have been ascribed by the patients wholly and completely to an injury received after they had reached adult years. Does not the fact throw much light on the error of writers and practitioners, who are so ready to admit the statements of patients that scirrhous and other tumours in the breast have been produced by local injuries?

*Cancer.* It is remarkable how fond numerous writers appear of the hypothesis, that the frequency of cancer in certain organs is to be accounted for by the vicissitudes and varying states of function to which those organs are exposed. Our author applies this theory (though, to do him justice, hesitatingly) to the explanation of the well-known frequency with which the mammary gland grows cancerous. The enlargement of the gland at puberty, its still further increase during pregnancy, the phenomena of lactation, the sympathetic swelling and tension of the organs during the menstrual period, and eventually their flaccidity and atrophy are all notable changes which cannot be without influence on disease. Plausible enough is argumentation of this stamp. But we can easily overturn it by availing ourselves of observations employed by Dr. Walshe in refuting a very similar, if not identical, doctrine put forward by another French writer in respect of the uterus. The ovaries are subjected to all the modes of change observed in the mammæ; some of them are even considerably more marked in the former than in the latter organ: now, while 1147 deaths occurred from cancer of the breast in ten years in Paris, 64 only were due to the ovary.

The author's chapter on cancer of the breast is judiciously written; but we have of late been obliged to accord so much space to the subject, that we can afford none on the present occasion; besides there is nothing in the present work which could prove novel to the readers of the late numbers of this Journal. But we must in a few words convey to our readers the practical results at which the author has arrived; they are in some respects, if not in all, distinctly accordant with those to which Dr. Walshe's investigations have led him, as referred to in a previous number. (Brit. and For. Med. Rev. No. XLII, p. 436.) Cancer may be cured by possibility by medical means; the operation may frequently be avoided; early operation is not advantageous, and may even be dangerous and hasten relapses; it should (if practised) be practised *at a late period, when every other means have failed.* This latter proposition is exactly that taught by Dr. Walshe also; we need scarcely remark how completely at variance it is with the common doctrine, according to which the knife cannot be used too early. We have perused the evidence put forward on this subject by our countryman especially with deep interest, and confess ourselves to have been startled at finding how fully our own experience, when closely sifted, confirms the opinions he puts forward. Thus, in speaking of extirpation of the cancerous testicle, Dr. Walshe says: "it must to all seeming prove a point of insurmountable difficulty to determine what cases are suitable for operation,—inasmuch as death has very speedily followed the use of the knife, when the general health was unimpaired, the tumour small, the skin free from morbid change, and the cord unaffected." (On Cancer, &c. p. 427.) A melancholy truth of which but too many examples rise in memory's sad array before us.

